# Basaloid Squamous Cell Carcinoma Involving the Orbit: A Case Report

**DOI:** 10.7759/cureus.92070

**Published:** 2025-09-11

**Authors:** Estefania Ramirez Marquez, Ricardo A Murati Calderon, José J López-Fontanet, Maria Correa, Joseph Campbell, Armando L Oliver

**Affiliations:** 1 Ophthalmology, University of Puerto Rico School of Medicine, Medical Sciences Campus, San Juan, PRI; 2 Department of Pathology and Laboratory Medicine, University of Puerto Rico, Medical Sciences Campus, San Juan, PRI

**Keywords:** basaloid squamous cell carcinoma, head and neck oncology, multidisciplinary management, ocular oncology, orbital mass

## Abstract

This report describes a case of an intraorbital basaloid squamous cell carcinoma (BSCC) that led to blindness in the affected eye and to severe deformation of the orbit. The patient, a 63-year-old man, presented with a two-month history of left periorbital pain and progressive vision loss in his left eye. His ocular history was pertinent for trauma to his left upper eyelid 10 years prior to presentation, resulting in a scar. He noted that the orbital tumor started with ulceration in the area of the scar two years prior to his presentation to our clinic. The patient had a history of a 40-pound weight loss and headaches in the two months prior to his evaluation. He delayed seeking treatment because of various social and mental health circumstances. An examination of the left orbit was remarkable for extensive erosion of the orbital rim, with crust, areas of necrosis, absence of orbital fat and soft tissue structures, absence of eyelids, injected conjunctiva, corneal conjunctivalization, and phthisic changes. A biopsy revealed squamous cell carcinoma with basaloid features. Subsequently, the patient was advised to undergo exenteration of the painful blind eye. He instead underwent systemic chemotherapy followed by radiation therapy. To the best of our knowledge, this is the first such documented case.

## Introduction

Basaloid squamous cell carcinoma (BSCC) is a rare, aggressive variant of squamous cell carcinoma (SCC), characterized histologically by a biphasic pattern of basaloid and squamous cell components [1-4]. On histopathology, the basaloid component typically forms compact nests, lobules, and trabeculae of tightly packed small cells with scant cytoplasm and hyperchromatic nuclei, often showing peripheral nuclear palisading, mitotic activity, central comedo-type necrosis, and microcystic spaces containing mucinous material [1,3-5]. The lesions associated with BSCC tend to affect the areas of the head and neck, specifically the upper aerodigestive tract [1-4]. This rare disease has an unknown etiology; nevertheless, most of the patients are reported to be about 60 years of age and to have a history of smoking and/or drinking alcohol [1-3].

Clinically, BSCC tends to present at an advanced stage and demonstrates aggressive behavior with regional nodal involvement and potential for distant metastasis, leading to worse outcomes than conventional SCC; reported survival is generally unfavorable across head-and-neck series [6]. Ophthalmic involvement is exceedingly uncommon. Ocular reports primarily describe conjunctival disease, whereas orbital involvement is usually secondary to direct extension from sinonasal primaries [7]. 

There is no standard treatment for managing BSCC; however, surgical resection, chemotherapy, and radiotherapy have been used with variable results [3,4,8,9]. The literature about BSCC is limited and heterogeneous; however, a timely diagnosis and monitoring are essential if a patient is to be provided with optimal management, as this condition may be life-threatening [2,4]. We hereby present the first documented case, to the best of our knowledge, of an intraorbital BSCC that led to blindness in the affected eye and to severe deformation of the orbit.

## Case presentation

A 63-year-old Hispanic man presented with a two-month history of periorbital pain and progressive vision loss in the left eye (OS). His past medical history was remarkable for bronchial asthma and major depressive disorder. His ocular history was pertinent for a trauma 10 years prior to presentation, which he described as a needle puncture through the left upper eyelid into the orbit. According to the patient, the globe was not penetrated, but he developed a scar in the left upper eyelid following the trauma. Ulceration in the area of the scar was noted by him about two years prior to his evaluation in our clinic. His review of systems was remarkable for having lost 40 pounds and intractable headaches in the two months leading up to his evaluation. The patient had a history of smoking cannabis and cigarettes, about nine pack-years. His family history included a son with gastric cancer. The patient delayed seeking treatment because of various social and mental health circumstances.

**Figure 1:**
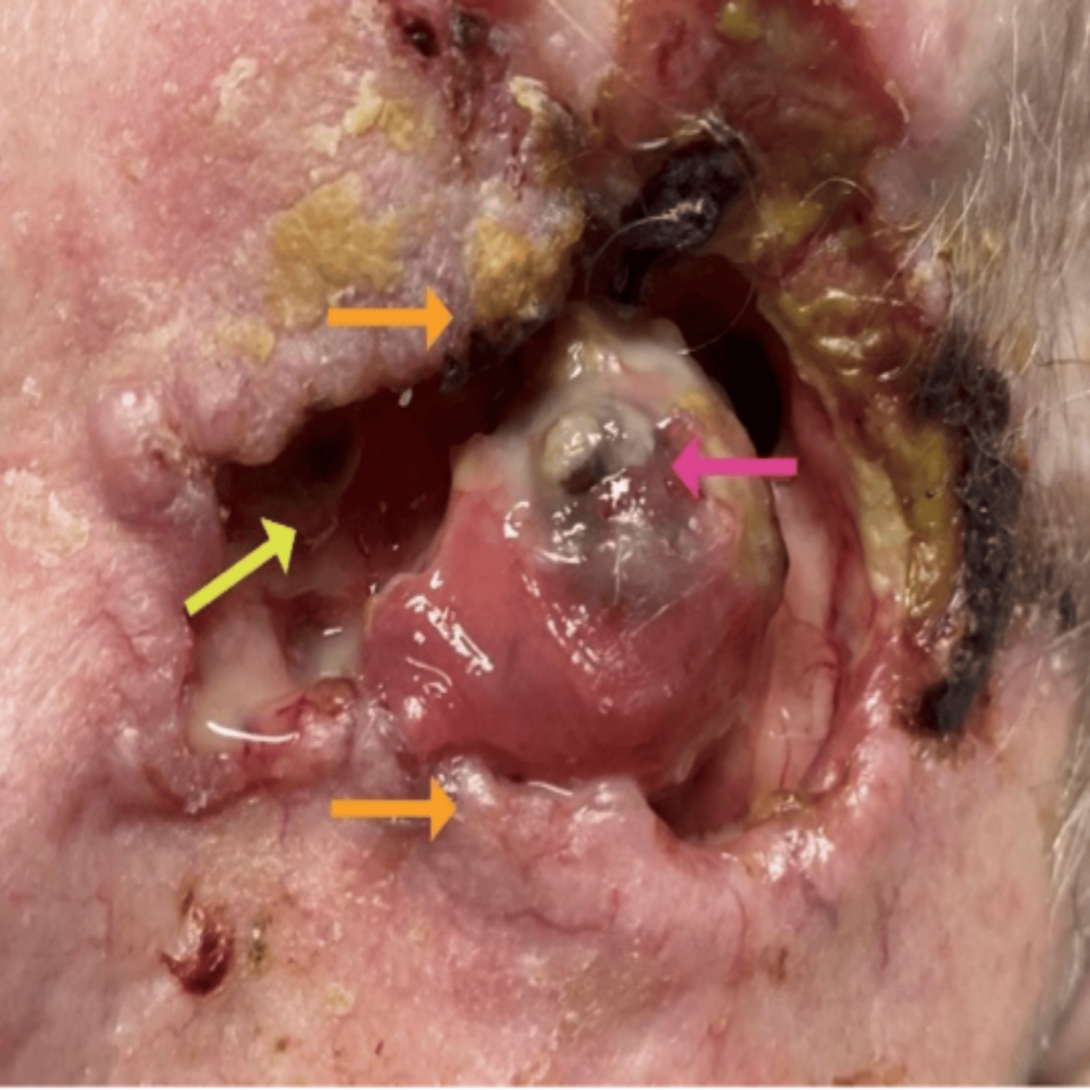
External view of the left orbit Eroded orbital rim with crusts and areas of necrosis (seen superotemporally) and a view of bone anatomy (yellow arrow). No orbital fat or soft tissue structures noted. Distortion of eyelids (orange arrows). Diffusely injected conjunctiva covering globe and corneal conjunctivalization with phthisic changes (pink arrow).

Upon comprehensive ophthalmic evaluation, his best-corrected visual acuity (BCVA) was 20/50, with pinhole improvement to 20/20, in the right eye (oculus dexter (OD)) and no light perception in the left eye (oculus sinister (OS)). The intraocular pressure was 10 mmHg in OD and could not be obtained in OS. The pupil was round and reactive to light in OD; however, there was no clear view to assess the OS. Color vision, as assessed by the Ishihara color plate test, revealed no defect in OD. Extraocular movements were within normal limits in OD, and there was 1mm of movement in all gazes in OS. A slit-lamp examination revealed erosion of the orbital rim (with crust), areas of necrosis, absence of orbital fat and soft tissue structures, absence of eyelids, injected conjunctiva, corneal conjunctivalization, and phthisic changes in OS (Figure 1); the OD was unremarkable. The patient's fundus was unremarkable in OD, and there was no view of the OS; therefore, no assessment could be made.

**Figure 2:**
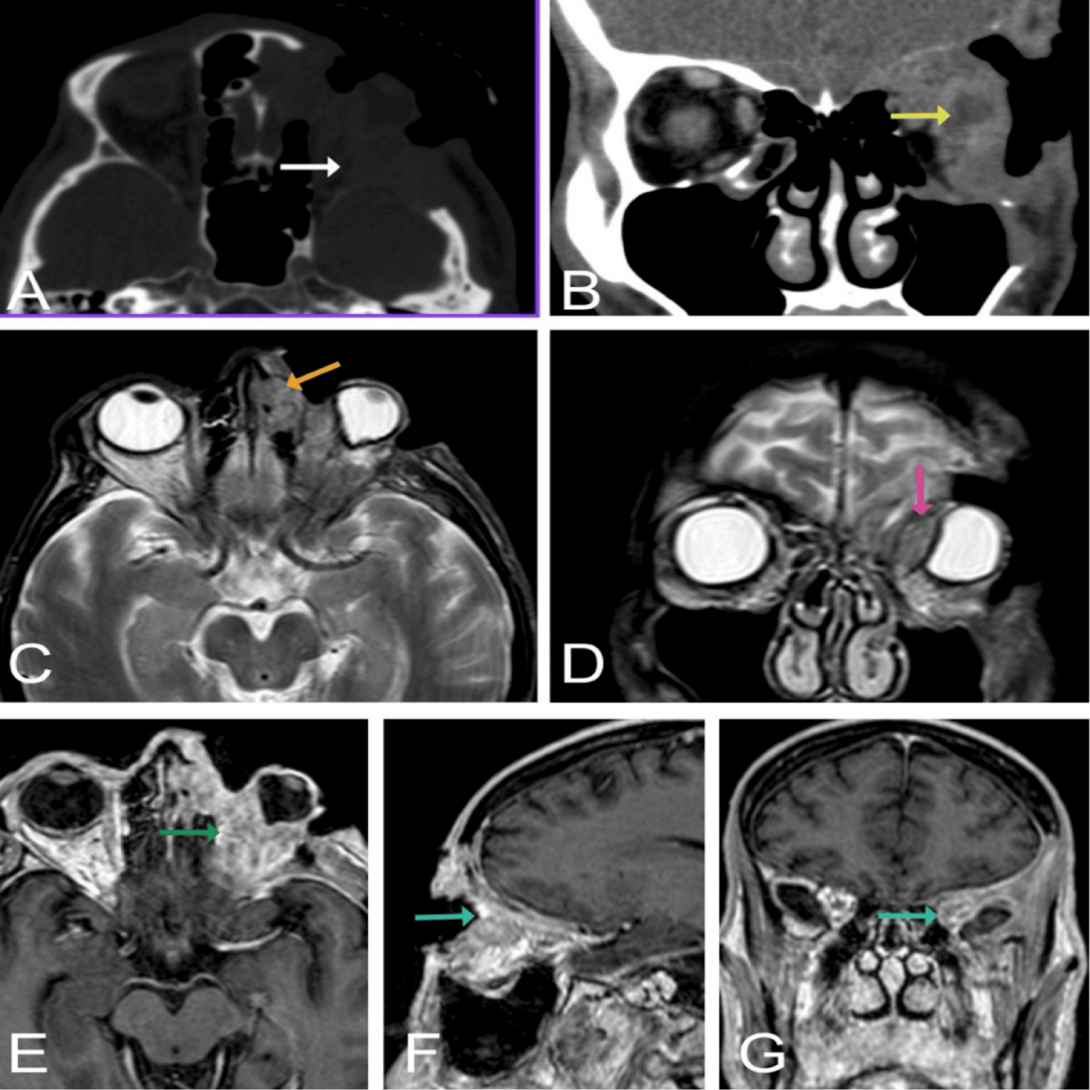
Orbital and facial computerized tomography (CT) and magnetic resonance imaging (MRI) Axial bone window (A) and coronal soft tissue window (B) in contrast-enhanced CT demonstrate a heterogeneously enhancing infiltrative soft tissue left orbital lesion causing mass effect on the left eye globe (yellow arrow), which appears deformed and compressed. There is additional infiltration of the extraocular muscles and osseous destruction/erosion of the left orbit, left maxillary sinus, left lamina papyracea and anterior left ethmoid air cells, left zygoma, left frontotemporal skull, and left sphenoid wing (white arrow). T2-weighted axial (C) and coronal (D) MRI demonstrate locally invasive heterogeneous T2 left orbital mass with obliteration of the extraconal fat, extension into the left anterior ethmoid air cells (orange arrow), and infiltration of the extraocular muscles with marked asymmetric enlargement of the left medial rectus muscle (pink arrow). Axial (E), sagittal (F), and coronal (G) post-contrast T1-weighted 3D MRI sequence demonstrate diffusely enhancing irregular aggressive appearing left orbital mass (green arrow) with osseous extension/infiltration as evidenced by abnormal enhancement of the left orbital walls including the roof and orbital apex (blue arrows), sphenoid bone, through the lamina papyracea into the ethmoid air cells, and left zygoma.  There was no evidence of intracranial extension, parenchymal involvement, or abnormal intracranial post-contrast enhancement.

**Figure 3:**
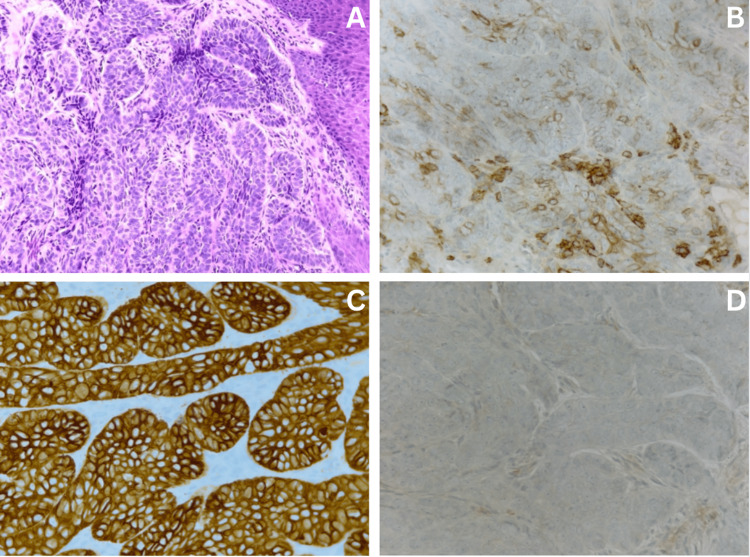
Histopathological slides (A) Nests of tumor cells with scant cytoplasm and peripheral palisading (H&E 20x). (B) Focal positivity for epithelial membrane antigen (EMA) (40x). (C) Tumor cells strongly and diffusely positive for CK5/6 (40x). (D) Negative expression for BerEP4 (40x).

An orbital computed tomography (CT) scan revealed extensive destruction of the left orbit and periorbital bones, obliteration of most of the conal fat, atrophic extraocular muscles, and no discrete enhancing lesion (Figure 2A, 2B). A brain magnetic resonance imaging (MRI) scan revealed an irregular soft tissue mass in the left orbit, characterized by localized and invasive behavior and a compressive effect causing left-globe deformity (Figure 2C-2G). Studies, including a complete blood cell count and a basic metabolic panel, were unremarkable. A biopsy disclosed SCC with basaloid features (Figure 3). Histology demonstrated basaloid nests with peripheral palisading and foci of necrosis. Immunohistochemistry confirmed a squamous cell phenotype with focal positivity for epithelial membrane antigen (EMA), strong diffuse CK5/6 positivity, and negative BerEP4. Taken together, the morphology and immunoprofile favored BSCC over basal cell carcinoma (BCC), the latter of which typically shows diffuse BerEP4 expression and EMA negativity with characteristic stromal retraction, findings not present in our specimen. 

The patient was advised to undergo exenteration of the left painful, blind eye; however, despite being informed about the associated benefits and risks, he declined the surgery. He was subsequently evaluated by the otolaryngology and neurosurgery services, who, from their respective specialties, determined that no additional surgical intervention was indicated at that time. He subsequently underwent a chemotherapy regimen consisting of an induction TPF (docetaxel-cisplatin-fluorouracil) for three cycles, followed by consolidation with concurrent carboplatin and cetuximab plus radiation therapy delivered in 12 fractions with good clinical response. At present, the patient is awaiting a post-chemoradiation positron emission tomography (PET) scan to assess treatment response and guide further management decisions in coordination with the otolaryngology service.

## Discussion

To our knowledge, this is the first documented case of an intraorbital BSCC leading both to blindness in the affected eye as well as to extensive orbital deformation. There have been only two reported cases of ocular BSCC, specifically affecting the conjunctiva without involvement of other intraorbital components [5,7]. Approximately 1% of SCCs are BSCCs [3]. Histopathologically, BSCC is distinguished by the presence of solid groups of cells arranged in a lobular configuration; small, crowded cells with scant cytoplasm; dark hyperchromatic nuclei without nucleoli; and small cystic spaces containing mucin-like material [1,3]. This rare disease has a predilection for the upper aerodigestive tract [2,3]. The average age of a person diagnosed with BSCC is around 60 years, and the majority of those affected are males [2]. Clinically, BSCC initially manifests as an exophytic lesion with ulceration; however, it tends to be diagnosed at advanced stages [3].

Across head-and-neck series, BSCC is reported to display a worse prognosis, characterized by rapid growth, early spread, and a low survival rate, relative to conventional SCC [10,11]. For example, in a matched case-control cohort, Soriano et al. found higher rates of distant metastasis in BSCC (45% vs 7%), with the lung being the most common site (71%), and distant disease accounting for the majority of fatalities (70% vs 13%) [10]. In line with these findings, a five-year disease-specific survival was lower in the BSCC group (36% vs 69%). Furthermore, nodal invasion at the time of diagnosis was associated with worse overall disease-free survival [10]. Site-specific population data in hypopharyngeal BSCC favor multimodality therapy, with surgery plus adjuvant radiation associated with more favorable disease-specific survival than other modalities [11]. For resectable head-and-neck BSCC, practice guidelines support primary surgery with elective neck dissection, followed by postoperative chemoradiotherapy, and recommend baseline positron emission tomography with biannual chest CT scans, given the predilection for early distant spread [11].

The diagnosis of BSCC is supported by several features in our specimen. Morphologically, basaloid nests with areas of comedo-type necrosis are consistent with basaloid squamous differentiation. Immunohistochemistry further supports this diagnosis: CK5/6 positivity confirms keratin expression, though it is not alone discriminatory; EMA positivity favors squamous differentiation and is rare in BCC; and BerEP4 negativity, which is classically positive in BCC but negative or only focal in BSCC, argues further against BCC. The absence of stromal retraction also disfavors BCC. Collectively, these findings are inconsistent with pure BCC and favor a diagnosis of BSCC.

Regarding other differentials, sebaceous carcinoma is less likely, as our specimen lacks sebaceous cytoplasmic vacuolization and does not express adipophilin or androgen receptor [12]. Poorly differentiated SCC can overlap morphologically, but the basaloid architecture with comedo-type necrosis in this case strongly supports the basaloid variant. Although not performed in our specimen, ancillary markers can further support the diagnosis. Diffuse nuclear p63/p40 expression substantiates squamous differentiation, and a high Ki-67 proliferative index is commonly observed in BSCC and less typically in BCC [13,14].

Smoking is regarded as a possible risk factor for the development of BSCC [1-3]. The patient described in this case did, in fact, have a long history of smoking, both cigarettes and cannabis. Interestingly, this patient also had a history of left-orbit trauma that led to scarring, and he identified the scar as being the site of the initial ulcerating lesion. It is possible that having a history of trauma may predispose to tumor development; however, to our knowledge, no prior cases have specifically linked trauma with BSCC. This association has only been reported in isolated cases of SCC and BCC [5,7]. Additionally, the patient having been associated with two completely separate lesions is also plausible. 

The rarity of BSCC and the limited data available describing it pose significant challenges for the clinical management of patients who suffer from this disease [1-4]. Ultimately, BSCC exhibits aggressive behavior and may have a poor prognosis, sometimes leading to a fatal outcome [15]. Some lesions may be amenable to resection, while others might need to be treated using radiotherapy, chemotherapy, or a combination of these approaches [4]. The extent of disease noted upon this patient’s presentation limited treatment options in this case. He was advised to undergo exenteration of the painful, blind eye; nevertheless, after a thorough discussion (with the surgeon) of the risks and benefits, he decided to postpone the exenteration. He continued to be followed up by various specialty services, such as otolaryngology, radiology, neurosurgery, and hematology-oncology. This holistic and multidisciplinary approach is essential for monitoring disease progression and the potential spread to critical structures, including the brain. Further studies should be conducted to promote the development of improved algorithms for treating the orbital involvement of BSCC.

## Conclusions

This report presented a case of intraorbital BSCC, leading to blindness in the affected eye as well as to orbital deformation. Smoking and prior trauma may be possible risk factors associated with this disease. Given the rarity of BSCC and the limited data about it, managing the disease poses challenges, but a comprehensive multidisciplinary approach may aid diagnosis, surveillance, and treatment planning. Clinicians should maintain a high index of suspicion for malignant transformation in atypical or changing periocular scars or nonhealing ulcers, and pursue early biopsy to avoid delays in definitive therapy. Given the scarcity of data, reporting additional cases with standardized clinical, imaging, and histopathologic details will help clarify risk factors and guide evidence-based management strategies. Ultimately, a patient-centered approach that balances cancer control with the preservation of vision and quality of life remains of key importance.
